# Functional analysis of the EsaB component of the *Staphylococcus aureus* Type VII secretion system

**DOI:** 10.1099/mic.0.000580

**Published:** 2017-11-22

**Authors:** M. Guillermina Casabona, Grant Buchanan, Martin Zoltner, Catriona P. Harkins, Matthew T. G. Holden, Tracy Palmer

**Affiliations:** ^1^​ Division of Molecular Microbiology School of Life Sciences, University of Dundee, Dundee, UK; ^2^​ School of Medicine, University of St Andrews, St Andrews, KY16 9TF, UK

**Keywords:** *Staphylococcus aureus*, protein secretion, T7SS, regulation

## Abstract

Type VII secretion systems (T7SS) are found in many bacteria and secrete proteins involved in virulence and bacterial competition. In *Staphylococcus aureus* the small ubiquitin-like EsaB protein has been previously implicated as having a regulatory role in the production of the EsxC substrate. Here we show that in the *S. aureus* RN6390 strain, EsaB does not genetically regulate production of any T7 substrates or components, but is indispensable for secretion activity. Consistent with EsaB being an essential component of the T7SS, loss of either EsaB or EssC are associated with upregulation of a common set of iron acquisition genes. However, a further subset of genes were dysregulated only in the absence of EsaB. Quantitative western blotting indicates that EsaB is present at very low levels in cells. Substitution of a highly conserved threonine for alanine or arginine resulted in a loss of EsaB activity and destabilisation of the protein. Taken together our findings show that EsaB is essential for T7SS activity in RN6390.

## Introduction

Protein secretion systems are nanomachines employed by bacteria to transport protein substrates across their cell envelopes. Gram-negative bacteria produce a number of different secretion machineries that export proteins involved in a wide variety of processes including signalling, nutrient scavenging, host interaction and virulence [1]. The Type VII secretion system (T7SS) is found in some Gram-negative and many Gram-positive bacteria, and is particularly common among organisms of the actinobacteria and firmicutes phyla [2]. The T7SS was initially described in the pathogenic mycobacteria *Mycobacterium tuberculosis* and *Mycobacterium bovis,* where the ESX-1 T7SS was shown to be essential for virulence, due to the secretion of two major T-cell antigens EsxA (formerly known as ESAT-6) and EsxB (formerly known as CFP-10) [3–5]. EsxA and EsxB are founding members of the WXG100 protein family that appear to be exclusively linked to T7SSs, and all characterised T7 systems are associated with at least one family member. The presence of a membrane-bound ATPase of the SpoIIIE/FtsK family (termed EccC in actinobacteria and EssC in firmicutes) is another hallmark of all T7SSs [6]. In Mycobacteria, three further membrane proteins EccB, EccD and EccE assemble with EccC to form a large 1.5 MDa core complex [7, 8]. This complex further associates with a membrane-bound mycosin serine protease, MycP, that is essential for T7 protein secretion and for stability of the membrane complex [9].

**Fig. 1.**
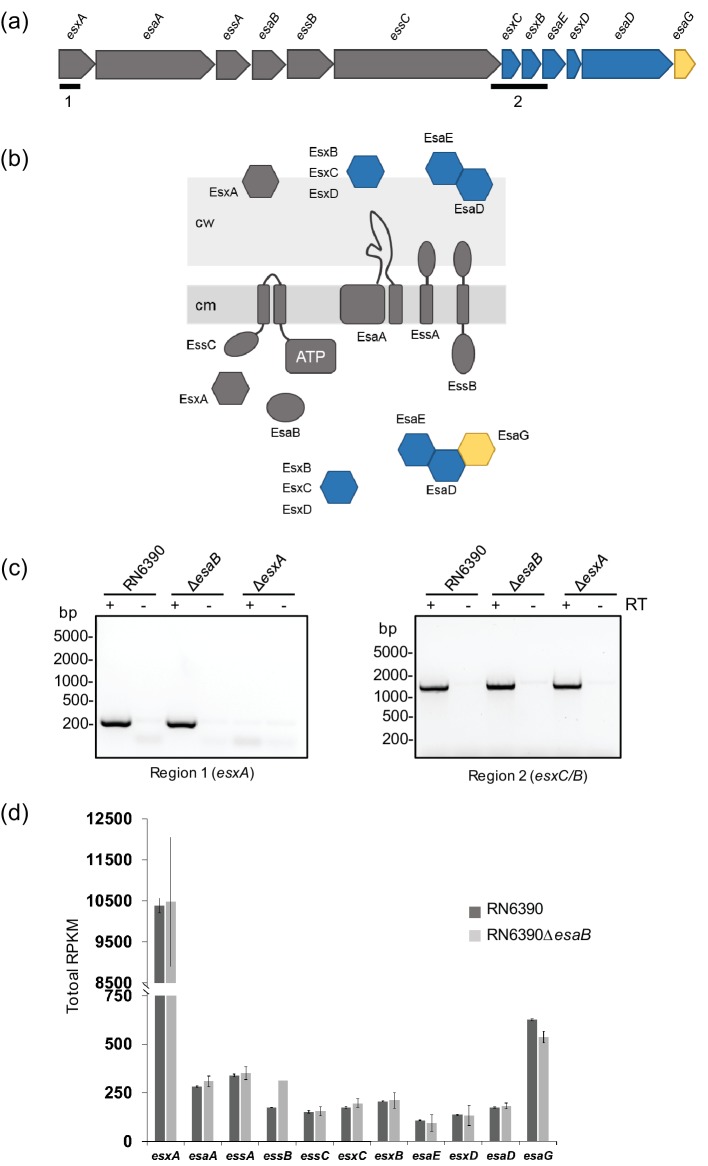
EsaB is not a transcriptional regulator. (a) The *ess* locus in *S. aureus* RN6390. Genes encoding essential secretion components are shaded in grey, secreted proteins in blue and a cytoplasmic antitoxin in yellow. The regions analysed by RT-PCR are indicated. (b) Predicted subcellular locations of Ess-encoded components. cw–cell wall, cm–cytoplasmic membrane. (c) RT-PCR analysis of *esxA* (region 1) and *esxC*/*B* (region 2) from the RN6390 and isogenic *esaB* and *esxA* mutant strains. Shading is as for Fig. 1(a) with essential secretion components in grey, secreted proteins in blue and a cytoplasmic antitoxin in yellow. Equivalent amounts of mRNA from each strain were used to generate cDNA. RT: reverse transcriptase. (d) Total mRNA counts of *ess* genes from RNA-Seq analysis of RN6390 and the *esaB* mutant strain. RPKM – reads of transcript per kilobase per million of mapped reads.

*Staphylococcus aureus*, an opportunistic pathogen of humans and animals, also elaborates a T7SS that is distantly related to the T7SSs found in mycobacteria [10]. Mutational analysis has indicated that it plays an important role in persistence in mouse models of infection, intra-species competition and potentially iron homeostasis [10–15]. In commonly-studied strains of *S. aureus* such as Newman, USA300 and RN6390, the secretion system is encoded by the 12 gene *ess* locus [10, 12, 16]. The first six genes at this locus encode essential components of the secretion machinery, including the WXG100 protein EsxA and the SpoIIIE/FtsK ATPase EssC (Fig. 1a, b). However, *S. aureus* and other firmicutes lack homologues of EccB, EccD, EccE and MycP and instead have an apparently unrelated set of membrane-bound secretion components (EsaA, EssA and EssB in *S. aureus*) [12, 17–19]. The sixth component of the *S. aureus* T7SS is EsaB, which is predicted to be a small cytoplasmic protein of 80 amino acids that is structurally related to ubiquitin [20]. In *S. aureus* strains Newman and USA300, a transposon insertion in *esaB* does not abolish secretion of T7 substrates but is linked with an increase in RNA transcripts covering the gene encoding the substrate EsxC [11]. By contrast, in-frame deletion of *esaB* abolished EsxA and EsxC secretion in strain RN6390 but did not detectably affect production of these substrate proteins [12]. Similarly, inactivation of *yukD*, which encodes the *Bacillus subtilis esaB* homologue, also abolished T7 secretion [17, 18].

In this study, we have addressed the role of EsaB in *S. aureus* T7 secretion using strain RN6390. We show that EsaB does not regulate *esxC* transcripts or those of other *ess*-encoded genes. Instead our findings show that EsaB behaves as an essential component of the T7SS. Interestingly, however, RNA-Seq analysis identified a subset of genes from the AirSR regulon that showed altered regulation in the absence of EsaB. This suggests that loss of *esaB* has additional unexpected effects on *S. aureus* physiology.

## Methods

### Bacterial strains and growth conditions

*S. aureus* strain RN6390 (NCTC8325 derivative, *rbsU*, *tcaR*, cured of φ11, φ12, φ13; [21]) and the isogenic Δ*esaB*, Δ*essC* and Δ*esx* (Δ*esxA – esaG*) strains [12] were employed in this study, along with strain Newman [22]. The *esaB* deletion strain is an in-frame deletion of the gene that maintains the first ten and final three codons of *esaB* (as there is a 9 codon overlap between the end of *essA* and the start of *esaB*). *S. aureus* strains were cultured in Tryptic Soy Broth (TSB) at 37 °C with shaking unless otherwise stated. For calculation of cell numbers we estimated by dilution analysis that one unit at OD 600 nm corresponds to 6×10^8^ c.f.u. for strain. When required, chloramphenicol (Cml, final concentration 10 µg ml^−1^) was added for plasmid selection. *E. coli* strain JM110 (Stratagene) was used for cloning purposes and BL21(DE3) [23] for EsaB overproduction and purification. *E. coli* was grown in Luria-Bertani (LB) medium at 37 °C with agitation. When appropriate, ampicillin was used for plasmid selection (final concentration 125 µg ml^−1^).

### Genetic constructs

All plasmids used in this study are listed in Table 1. The *esaB* gene with its own RBS was PCR amplified from *S. aureus* RN6390 genomic DNA using primers EsaB-fw and EsaB-rev (Table S1, available with the online version of this article). The 0.3 kb *Hpa*I/*Eco*RI restriction fragment was cloned into pRAB11 under control of the tetracycline inducible promoter, giving pRAB11-esaB. Clones were selected in *E. coli* and verified by DNA sequencing. Plasmid pRAB11-esaB-YFP was generated by cloning the 0.3 kb *Hpa*I/*Eco*RI restriction fragment into pRAB11-YFP [15]. Clones were selected in *E. coli* and verified by DNA sequencing. Nucleotide variants of *esaB* were generated by the Quickchange site-directed mutagenesis protocol (Stratagene) using pRAB11-esaB or pRAB11-esaB-YFP as a template and primers listed in Table S1. Modified plasmids were digested using *Dpn*I for at least 1 h at 37 °C and transformed into *E. coli*. Single point mutations were verified by DNA sequencing.

**Table 1.** Plasmids used in this study

Plasmid	Relevant genotype or description	Source or reference
pRAB11	*E. coli/S. aureus* shuttle vector, inducible protein expression, Amp^r^, Cml^r^	[35]
pRAB11-esaB	pRAB11 producing EsaB	This study
pRAB11-esaB-T8A	pRAB11 producing T8A-substituted EsaB	This study
pRAB11-esaB-T8E	pRAB11 producing T8E-substituted EsaB	This study
pRAB11-esaB-T8R	pRAB11 producing T8R-substituted EsaB	This study
pRAB11-esaB-T8H	pRAB11 producing T8H-substituted EsaB	This study
pRAB11-esaB-T8K	pRAB11 producing T8K-substituted EsaB	This study
pRAB11-esaB-T8S	pRAB11 producing T8S -substituted EsaB	This study
pRAB11-esaB-D10A	pRAB11 producing D10A-substituted EsaB	This study
pRAB11-esaB-D20A	pRAB11 producing D20A-substituted EsaB	This study
pRAB11-esaB-L21A	pRAB11 producing L21A-substituted EsaB	This study
pRAB11-esaB-K30A	pRAB11 producing K30A-substituted EsaB	This study
pRAB11-esaB-K52A	pRAB11 producing K52A-substituted EsaB	This study
pRAB11-esaB-K56A	pRAB11 producing K56A-substituted EsaB	This study
pRAB11-esaB-L66A	pRAB11 producing L66A-substituted EsaB	This study
pRAB11-esaB-G74A	pRAB11 producing G74A-substituted EsaB	This study
pRAB11-esaB-D75A	pRAB11 producing D75A-substituted EsaB	This study
pRAB11-esaB-YFP	pRAB11 producing EsaB-YFP	This study
pRAB11-esaB-T8A-YFP	pRAB11 producing T8A-substituted EsaB-YFP	This study
pRAB11-esaB-T8R-YFP	pRAB11 producing T8R-substituted EsaB-YFP	This study
pET15b-HISEsaB	pET15b expressing 6XHis-tagged EsaB	This study

### RNA isolation and RT-PCR

For RNA-Seq analysis, three biological repeats of the *S. aureus esaB* strain was grown aerobically in TSB up to an OD_600_ of 1 at which point mRNA was prepared (in three technical replicates). This experiment was carried out alongside the RN6390 and *essC* strains [15] and followed identical methodology.

For RT-PCR, the indicated *S. aureus* strains were grown aerobically in TSB and harvested at an OD_600_ of 1. At this point, the mRNA was extracted using the SV total RNA Isolation Kit (Promega) with some minor modifications. Cell samples were stabilised in 5 % phenol/95 % ethanol on ice for at least 30 min and then centrifuged at 2770 ***g*** for 10 min. Cells were then resuspended in 100 µl of TE buffer containing 500 µg ml^−1^ lysostaphin and 50 µg ml^−1^ lysozyme and incubated at 37 °C for 30 min. Subsequently, the manufacturer’s instructions were followed. Isolated RNA was subjected to a second DNase treatment using the DNA-free kit (Ambion). RNA was stored at −80 °C until use. RT-PCR to probe transcription of genes in the indicated strains was carried out using 500 ng of mRNA as template with the indicated primers (Table S1). PCR products were visualized on 1 % agarose gels.

### Purification of 6His-EsaB and generation of polyclonal antisera

The EsaB coding sequence (UniProt code ESAB_STAAM) was PCR amplified from a synthetic gene (codon optimised for *E. coli* K12 (Genscript)) using the primers EsaB-pET1 and EsaB-pET2 (Table S1) and cloned into the *Nde*I*/Xho*I site of a modified pET15b vector (Novagen). The plasmid produces an N-terminal His_6_-tagged protein with a TEV (tobacco etch virus) protease cleavage site. The protein was expressed and purified as described previously [24], except the tag-free EsaB was not collected in the flow-through of the negative purification but required a 30 mM imidazole elution. The final size exclusion chromatography step used a 24 ml HR 30/100 GL Superdex75 column (GE healthcare), equilibrated with 20 mM Tris pH 7.8, 100 mM NaCl and was calibrated with molecular mass standards (thyroglobulin, 670 kDa; γ-globulin, 158 kDa; serum albumin, 67 kDa; ovalbumin; 44 kDa, myoglobin, 17 kDa; and vitamin B12, 1 kDa). Two mg purified EsaB (retaining a Gly–Ala–Ser–Thr sequence at the N-terminus after the cleavage step) was utilised as antigen to immunise rabbits for polyclonal antibody production in a standard three injections protocol (Seqlab).

### Secretion assays, subcellular fractionation and western blotting

The indicated strains were grown overnight in TSB, diluted 1/100 in fresh medium and grown up to mid-log phase, at which point whole cells and supernatant fractions were harvested as described previously [12]. Briefly, cells and supernatant were separated by a 10 min centrifugation step at 2770 ***g***. Cells were washed twice with PBS, adjusted to and OD_600_ of 1 and digested using 50 µg ml^−1^ of lysostaphin by incubation at 37 °C for 30 min. Supernatants were filtered using a 0.22 µm filter and TCA-precipitated in the presence of 50 µg ml^−1^ deoxycholate, as described. For *S. aureus* subcellular fractionation, cells were grown to mid-log phase with shaking and treated as previously described [12]. Briefly, cells were harvested by centrifugation and resuspended in TSM buffer (50 mM Tris-HCl pH 7.6, 0.5 M sucrose, 10 mM MgCl_2_). Lysostaphin was added to a final concentration of 50 µg ml^−1^ and cells were incubated at 37 °C for 30 min to digest the cell wall. At this point, protoplasts were sedimented to recover the cell wall (supernatant fraction). Protoplasts were disrupted by sonication and the membrane was obtained after an ultracentrifugation step at 227 000 ***g*** for 30 min and at 4 °C. The supernatant was retained as the cytoplasmic fraction. Samples were boiled for 10 min prior to separation in bis-Tris gels and subsequent western blotting.

Polyclonal antisera were used at the following dilutions: α-EsxA 1 : 2500 [12], α-EsxB 1 : 1000 [15], α-EsxC 1 : 2000 [12], α-EsaB 1 : 500, α-TrxA 1 : 20 000 [25] and α-SrtA (Abcam) 1 : 3000. Anti-GFP antibody was obtained from Roche and used according to manufacturer’s instructions.

## Results

### EsaB does not regulate the level of *esxC* transcripts in strain RN6390

A previous study has shown that a transposon insertion in the *esaB* gene results in an increase in *esxC* transcripts in the Newman and USA300 strain backgrounds, and a concomitant increase in the EsxC polypeptide, implicating it as a regulator [11]. To investigate whether loss of *esaB* by in-frame deletion affects the level of *esxC* mRNA in strain RN6390, we isolated mRNA from the parental strain and the isogenic *esaB* mutant, prepared cDNA and undertook reverse transcriptase PCR with primers covering either *esxA* (the first gene at the *ess* locus, included as a negative control) or *esxC* (Fig. 1a). It can be seen (Fig. 1c) that the level of transcripts for each of these genes was qualitatively similar in the wild-type and *esaB* backgrounds.

To examine this quantitatively, we undertook RNA-Seq analysis on RNA prepared from three biological repeats of the RN6390 and *esaB* strains grown aerobically in TSB to an OD_600_ of 1. Note that these experiments were performed at the same time as the RN6390 versus *essC* RNA-Seq analysis described in [15] and used the same RN6390 dataset. Fig. 1(d) shows that the level of *esxC* transcripts were indistinguishable between the wild-type and *esaB* strains. Analysis of the transcript levels of the other genes at the *ess* locus indicates that in general they were also not significantly altered by the loss of *esaB* although there was a small increase in the level of *essB*. We conclude that there is no evidence that *esaB* regulates the level of *esxC* transcripts in RN6390.

**Fig. 2.**
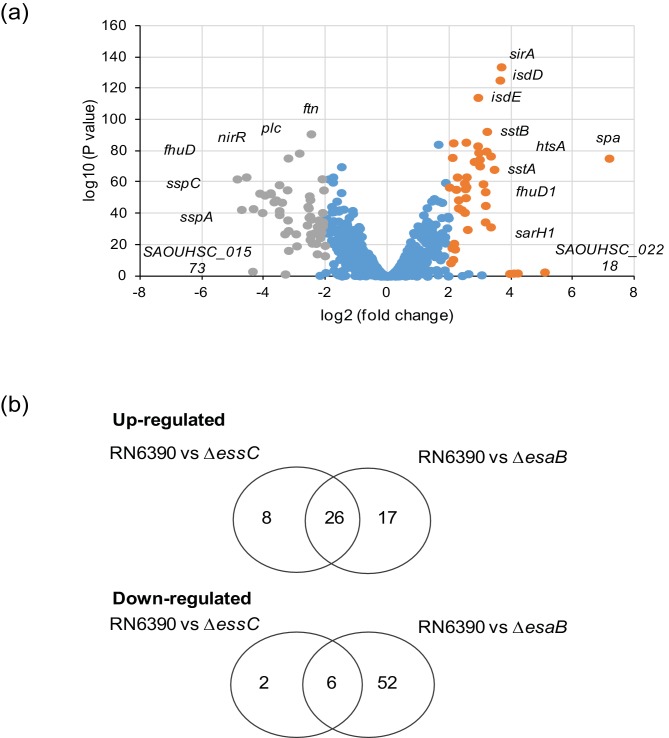
RNA-Seq analysis of differentially regulated genes in the *esaB* mutant strain. (a) Volcano plot representation of the differentially expressed genes in RN6390 strain compared to the isogenic *esaB* mutant. The orange and grey spots represent, respectively, genes that are up- or down-regulated in the *esaB* mutant relative to the parental strain. (b). Overlap between up- and down-regulated genes in the *esaB* and *essC* datasets.

**Fig. 3.**
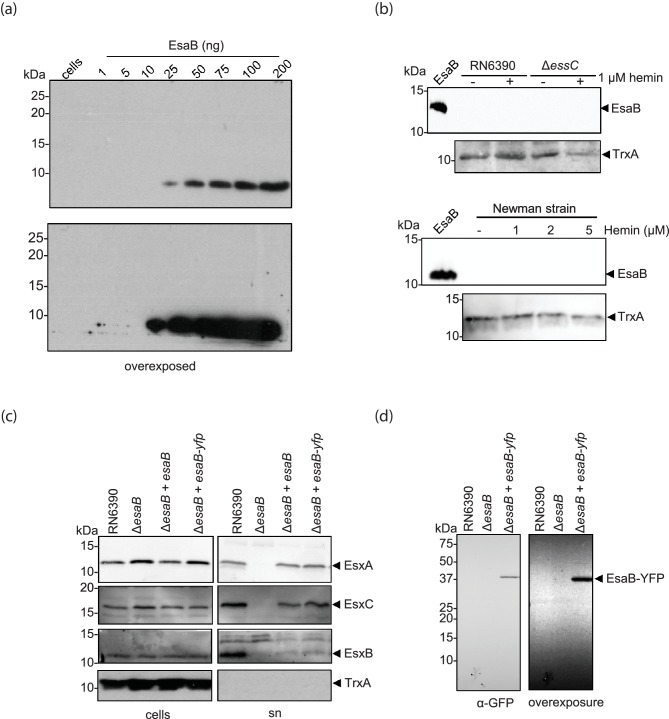
EsaB-YFP localises to the cytoplasm and membrane. (a) EsaB-YFP is not secreted in *S. aureus* strain RN6390. RN6390 harbouring empty pRAB11 and the isogenic Δ*esaB* strain harbouring empty pRAB11 or pRAB11 encoding EsaB-YFP were cultured in TSB medium until mid-log phase and separated into cellular and supernatant fractions (sn). For each gel, 10 µl of OD_600_1 adjusted cells and 15 µl of TCA-precipitated culture supernatant were loaded. Blots were probed with anti-EsxA, anti-TrxA (cytoplasmic control) and anti-GFP antisera. Cell and supernatant samples have been blotted on the same gel but intervening lanes have been spliced out. Subcellular localisation of (b) EsaB-YFP in RN6390 and an isogenic Δ*esx* (Δ(*esxA-esaG*)) strain or (c) YFP in RN6390. Cells were grown aerobically in TSB to mid-log phase and fractionated as indicated in the Methods. Equivalent amounts of each fraction was probed with anti-TrxA (cytoplasmic control), anti-SrtA (membrane control), anti-EsxA and anti-GFP antisera.

We next examined the entire transcript profile of the *esaB* mutant to investigate the transcriptional/post-transcriptional response to the loss of this small protein. We found 101 genes de-regulated in the *esaB* mutant compared to the parental strain (using a cut off of logFC >2 or <−2 and qvalue <0.05, as applied previously [15]), Fig. 2(a). Of these, 43 were upregulated by the loss of *esaB* whereas 58 were downregulated when *esaB* was absent – these genes are listed in Table 2. Interestingly, almost all of the genes that were differentially regulated in the *essC* mutant [15] were also similarly regulated in the *esaB* strain (Fig. 3b), although there was a substantive subset of genes that were differentially expressed in the *esaB* mutant but not the *essC* strain (Table 2). It can be seen that almost all of the iron acquisition genes, including those for heme acquisition, staphyloferrin synthesis and uptake and ferrichrome import were commonly upregulated by loss of either *esaB* or *essC* (Table 2). Furthermore six of the eight downregulated genes from the *essC* strain were also down regulated in the *esaB* strain (note that one of the two genes unaffected in the *esaB* dataset is *essC* itself, which appears downregulated in the *essC* dataset because it has been deleted). The finding that almost the entire subset of genes differentially regulated in the absence of *essC* is also similarly altered by loss of *esaB* strongly suggests that EsaB is, like EssC, a component that is essential for activity of the secretion machinery in strain RN6390.

**Table 2.** Genes differentially regulated (>log 2 fold) in the RN6390 *esaB* deletion mutant, sorted by ascending fold change (FC) Genes highlighted in grey are also differentially regulated in the *essC* deletion strain. The column on the right shows the fold change (FC) of the same gene in the *essC* dataset where ns indicates no statistically significant change in expression level relative to the same gene in the wild-type dataset.

Locus ID	Gene name	FC in *esaB* mutant	Proposed function	FC in *essC* mutant
Downregulated genes				
SAOUHSC_00986	*sspC*	−23.7	Cysteine protease	ns
SAOUHSC_00988	*sspA*	−22.3	Glutamyl endopeptidase	ns
SAOUHSC_00987	*sspB*	−20.8	Cysteine protease	ns
SAOUHSC_01573	–	−19.0	Unknown, hypothetical protein	ns
SAOUHSC_01941	*splB*	−18.8	Serine protease SplB	−4.3
SAOUHSC_02971	*aur*	−17.1	Zinc metalloproteinase aureolysin	ns
SAOUHSC_01942	*splA*	−16.4	Highly specific serine protease specific to *S. aureus*	−5.4
SAOUHSC_02680	*narH*	−15.7	Nitrate reductase subunit beta	ns
SAOUHSC_01944	–	−14.3	Unknown, hypothetical protein	−4.5
SAOUHSC_02681	*narG*	−14.3	Nitrate reductase subunit alpha	ns
SAOUHSC_01121	*hla*	−13.5	α-hemolysin	−4.1
SAOUHSC_02241	*lukF*	−13.0	Unknown, hypothetical protein	−3.3
SAOUHSC_02163	*hlb*	−12.3	β-hemolysin	ns
SAOUHSC_01938	*splD*	−12.2	Serine protease SplD	−4.3
SAOUHSC_02679	*narJ*	−12.2	Nitrate reductase subunit delta	ns
SAOUHSC_02671	*narK*	−11.6	Putative nitrate transporter	ns
SAOUHSC_02455	*lacA*	−11.0	Galactose-6-phosphate isomerase subunit LacA	nd
SAOUHSC_01530	–	−10.9	Hypothetical phage protein	ns
SAOUHSC_01542	–	−10.9	Unknown, SNF2 family protein	ns
SAOUHSC_01535	–	−10.9	Phage capsid protein	ns
SAOUHSC_02240	*hlb*	−10.5	Truncated β-hemolysin	ns
SAOUHSC_02243	*lukG*	−10.4	Leukocidin like toxin	−4.5
SAOUHSC_02685	*nirR*	−10.3	Unknown, hypothetical protein	ns
SAOUHSC_01939	*splC*	−10.3	Serine protease SplC	−3.2
SAOUHSC_01937	–	−10.3	Unknown, hypothetical protein	−2.8
SAOUHSC_02970	*argR*	−8.8	Arginine repressor family protein	ns
SAOUHSC_00113	*adhE*	−8.6	Bifunctional acetaldehyde-CoA/alcohol dehydrogenase	ns
SAOUHSC_00051	*plc*	−8.1	1-phosphatidylinositol phosphodiesterase	−2.5
SAOUHSC_00898	*argH*	−6.7	Argininosuccinate lyase	ns
SAOUHSC_02684	*nasD*	−6.6	Assimilatory nitrite reductase [NAD(P)H] large subunit	ns
SAOUHSC_02709	*hlgC*	−6.5	γ-hemolysin component C precursor	−1.8
SAOUHSC_02682	*nasF*	−6.4	Uroporphyrin-III C-methyltransferase	ns
SAOUHSC_02462	–	−6.4	Unknown, hypothetical protein	ns
SAOUHSC_00401	–	−6.3	Putative exported protein	−1.6
SAOUHSC_01950	*epiD*	−6.3	Flavoprotein	ns
SAOUHSC_01936	*splE*	−6.3	Serine protease SplE	−3.3
SAOUHSC_02454	*lacB*	−6.3	Galactose-6-phosphate isomerase subunit LacB	−3.4
SAOUHSC_00899	*argG*	−6.2	Argininosuccinate synthase	ns
SAOUHSC_02108	*ftn*	−6.1	Ferritin	ns
SAOUHSC_00368	–	−6.1	Unknown, hypothetical protein	ns
SAOUHSC_00411	–	−5.9	Unknown, hypothetical protein	−2.2
SAOUHSC_01951	*epiC*	−5.8	Epidermin biosynthesis protein EpiC	ns
SAOUHSC_02683	*nasE*	−5.6	Assimilatory nitrite reductase [NAD(P)H] small subunit	ns
SAOUHSC_01935	*splF*	−5.3	Serine protease SplF	−2.7
SAOUHSC_02452	*lacD*	−5.2	Tagatose 1,6-diphosphate aldolase	−2.6
SAOUHSC_01953	*epiA*	−5.2	Gallidermin superfamily EpiA protein	ns
SAOUHSC_02941	*nrdG*	−4.9	Anaerobic ribonucleotide reductase activating protein	ns
SAOUHSC_00717	*saeP*	−4.7	Putative lipoprotein	−1.4
SAOUHSC_01990	*glnQ*	−4.6	Glutamine transport ATP-binding protein	ns
SAOUHSC_02557	*yut*	−4.5	Putative urea transporter	ns
SAOUHSC_01949	*epiP*	−4.4	Intracellular serine protease	ns
SAOUHSC_00120	capG	−4.4	UDP-N-acetylglucosamine 2-epimerase	ns
SAOUHSC_01952	*bsaB*	−4.4	Lantibiotic epidermin biosynthesis protein EpiB	ns
SAOUHSC_03015	*hisZ*	−4.4	ATP phosphoribosyltransferase regulatory subunit	ns
SAOUHSC_00119	*capF*	−4.4	Capsular polysaccharide biosynthesis protein CapF	ns
SAOUHSC_02463	*hysA*	−4.3	Hyaluronate lyase	ns
SAOUHSC_02453	*lacC*	−4.1	Tagatose-6-phosphate kinase	−2.2
SAOUHSC_00608	*adh1*	−4.1	Alcohol dehydrogenase	ns
Upregulated genes				
SAOUHSC_02767	*opp-1A*	4.0	Peptide ABC transporter substrate-binding protein	2.6
SAOUHSC_02655	–	4.2	Unknown, hypothetical protein	6.3
SAOUHSC_01292	–	4.4	Putative DNA-binding protein	ns
SAOUHSC_00130	*isdI*	4.4	Heme-degrading monooxygenase IsdI	5.7
SAOUHSC_00176	–	4.5	Extracellular solute-binding protein	ns
SAOUHSC_02435	*sfaA*	4.5	Putative transporter	6.7
SAOUHSC_02799	*sarT*	4.6	Staphylococcal accessory regulator	ns
SAOUHSC_02432	–	4.8	Unknown, hypothetical protein	6.2
SAOUHSC_02245	–	4.9	Unknown, hypothetical protein	6.5
SAOUHSC_00652	*fhuA*	5.1	Ferrichrome ABC transporter ATP-binding protein FhuA	7.0
SAOUHSC_00071	*sirC*	5.3	Involved in staphyloferrin B transport into the cytoplasm	4.6
SAOUHSC_00131	–	5.3	Putative membrane protein	6.1
SAOUHSC_02821	–	5.8	Putative membrane protein	ns
SAOUHSC_02719	–	6.2	ABC transporter ATP-binding protein	5.5
SAOUHSC_01920	–	6.3	Putative lipoprotein	ns
SAOUHSC_02428	*htsB*	6.3	Heme transport system permease HtsB	5.4
SAOUHSC_00974	–	6.4	Unknown, hypothetical protein	ns
SAOUHSC_01081	*isdA*	6.5	Iron-regulated heme-iron binding protein	5.4
SAOUHSC_00072	*sirB*	6.5	Involved in staphyloferrin B transport into the cytoplasm	7.4
SAOUHSC_02554	*fhuD2*	6.5	Ferric hydroxamate receptor 1 FhuD2	6.8
SAOUHSC_01090	–	6.7	Unknown, hypothetical protein	3.9
SAOUHSC_00973	–	7.9	Unknown, hypothetical protein	ns
SAOUHSC_01086	*isdF*	8.5	ABC permease IsdF	6.1
SAOUHSC_01085	*isdE*	8.6	Heme-receptor lipoprotein IsdE	5.6
SAOUHSC_01089	*isdG*	8.7	Heme-degrading monooxygenase IsdG	4.7
SAOUHSC_01087	–	8.9	Iron compound ABC transporter permease	6.3
SAOUHSC_01082	*isdC*	8.9	Heme transporter IsdC	5.5
SAOUHSC_00748	*sstC*	9.6	Ferrichrome ABC transporter ATP-binding protein SstC	9.1
SAOUHSC_00545	*sdrD*	10.0	Fibrinogen-binding protein SdrD	ns
SAOUHSC_02246	*fhuD1*	10.0	Iron compound ABC transporter FhuD1	8.0
SAOUHSC_00972	–	10.1	Unknown, hypothetical protein	ns
SAOUHSC_01088	*srtB*	10.2	Sortase StrB	6.2
SAOUHSC_00747	*sstB*	10.4	Ferrichrome ABC transporter permease SstB	9.0
SAOUHSC_00070	*sarH1*	11.2	Unknown, hypothetical regulatory-like protein	ns
SAOUHSC_02430	*htsA*	11.2	Heme transport system lipoprotein HtsA	10.5
SAOUHSC_00746	*sstA*	11.9	Ferrichrome ABC transporter permease SstA	10.9
SAOUHSC_01084	*isdD*	13.3	ATP-hydrolysing and heme-binding protein IsdD	6.2
SAOUHSC_00074	*sirA*	13.6	Receptor component of staphyloferrin B	16.3
SAOUHSC_01514	–	15.6	Unknown, hypothetical protein	ns
SAOUHSC_02232	–	16.7	Hypothetical phage protein	ns
SAOUHSC_02084	–	17.7	Phage repressor protein	ns
SAOUHSC_02218	–	25.9	Unknown, hypothetical protein	ns
SAOUHSC_00069	*spa*	51.5	Protein A	ns

As mentioned above, a subset of transcripts were differentially expressed in the *esaB* but not the *essC* strain. These include downregulated genes required for anaerobic nitrate respiration (*narGHJ*/*narK*), some secreted proteases (*sspA/B/C*, *aur*), capsular polysaccharide synthesis (*capG/F/hysA*), lactose metabolism (*lacB/C/D*) and antimicrobial peptide synthesis (*epiA/C/D/P*). Many of these genes are under control of the essential two component regulatory system AirSR (formerly YhcSR) [26–29]. This observation indicates that EsaB has additional effects on *S. aureus* physiology. This could be indirect and arising from its role in T7 secretion, for example through altered membrane permeability when EsaB is absent. Alternatively, EsaB may have additional roles in the cell in addition to its requirement for T7 protein secretion.

### EsaB is present at low amounts in cells of *S. aureus* RN6390

**Fig. 4.**
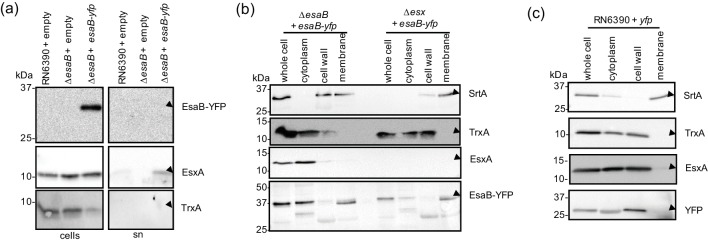
EsaB is present in cells at low amounts. (a) Titration of α-EsaB antibodies. The indicated amounts of purified EsaB, alongside 30 µl of OD_600_5 adjusted cells were loaded on a SDS-PAGE as indicated and blotted using α-EsaB antibodies. Two exposures of the blot are shown. (b) RN6390 and the isogenic Δ*essC* strain (top) or strain Newman (bottom panel) were grown aerobically in TSB medium with or without hemin, as indicated, until an OD_600_ of 2 was reached, at which point cells were harvested as described in Methods. In each case, for detection of EsaB, 25 µl of OD_600_2 adjusted cells were loaded and 25 ng of purified EsaB protein was loaded as a positive control. 5 µl of OD_600_2 adjusted cells were probed against anti-TrxA antisera as a cytoplasmic control. (c) RN6390 harbouring empty pRAB11 (labelled RN6390), and the isogenic *esaB* deletion strain harbouring pRAB11 (labelled Δ*esaB*), or pRAB11 encoding native EsaB or EsaB-YFP was cultured aerobically in TSB medium until an OD_600_ of 2 was reached. Samples were fractionated to give cells and supernatant (sn), and supernatant proteins were precipitated using TCA. For each gel, 10 µl of OD_600_1 adjusted cells and 15 µl of culture supernatant were loaded. Blots were probed with anti-EsxA, anti-EsxB or anti-EsxC antisera, alongside anti-TrxA (cytoplasmic control). Cell and supernatant samples have been blotted on the same gel but intervening lanes have been spliced out. (d) EsaB-YFP can be detected in whole cells. RN6390 harbouring empty pRAB11 (labelled RN6390), and the isogenic *esaB* deletion strain harbouring pRAB11 (labelled Δ*esaB*), or pRAB11 encoding EsaB-YFP was cultured aerobically in TSB medium until an OD_600_ of 2 was reached. Whole cell samples (20 µl of OD_600_2 adjusted cells) were loaded and blots were probed with anti GFP antibodies. Two exposures of the blot are shown.

To explore the biological role of EsaB in T7 secretion, we overproduced recombinant EsaB with a cleavable His-tag in *E. coli*. The purified protein eluted from gel filtration as a monomer, in agreement with structural analysis of the *B. subtilis* EsaB homologue, YukD, which also appears to be monomeric [20]. Polyclonal antisera were raised against purified EsaB and the antibody was affinity purified against the EsaB antigen, before being used to detect the protein in whole cells of *S. aureus*. Fig. 4(a) shows that although the purified antiserum could clearly recognise purified EsaB, it did not detect a band of the expected size of EsaB in whole cells. We have shown previously that expression of the T7SS genes in RN6390 is upregulated approximately 2–3-fold in the presence of exogenous hemin, and fourfold by hemin in a Δ*essC* background [15]. However, supplementation of either of these strains with hemin did not result in detectable EsaB in the cellular fraction (Fig. 4b) and it could also not be detected in cells of strain Newman (Fig. 4b). Probing a dilution series of purified EsaB indicated that the antibody was able to cross-react with as little as 25 ng of protein (Fig. 4a), which is equivalent to 1.6×10^11^ EsaB molecules. Since the antibody was unable to detect EsaB in whole cells of RN6390 from 9.6×10^8^ colony forming units that were loaded onto the SDS gel, we conclude that are less than 170 molecules of EsaB per cell.

Since we were unable to detect native EsaB in *S. aureus* cell extracts, we constructed a series of tagged variants for which commercial antisera were available. To this end we introduced His_6_, Myc, hemagglutinin (HA) and Strep epitopes onto the N-terminus of EsaB, and His_6_, Myc, HA, mCherry or FLAG epitopes onto the C-terminus, but in each case were unable to detect the tagged protein (not shown). We also introduced His_6_ and His_9_ epitopes into two predicted loop regions internal to the EsaB sequence but again were unable to detect tagged EsaB (not shown). The only tag we introduced that allowed detection of EsaB was a C-terminal yellow fluorescent protein (YFP) tag. Fig. 4(c) shows that basal production of either native (untagged) EsaB or EsaB-YFP from plasmid vector pRAB11 was sufficient to restore secretion of the T7SS extracellular protein EsxA and of substrates EsxB and EsxC to the culture supernatant. Blotting the same cell samples for the presence of the YFP fusion protein (Fig. 4d) showed that it migrated at close to the predicted mass (37 kDa) of the EsaB fusion. There was no evidence for degradation of the fusion protein even after prolonged exposure of the immunoblot (Fig. 4d). We conclude that the YFP-tagged variant of EsaB probably retains functionality.

### EsaB-YFP partially localises to the cell membrane

EsaB is predicted to be a soluble cytoplasmic protein [10], and is known to share structural homology with ubiquitin [20]. Interestingly, a domain sharing the same fold is also associated with the actinobacterial T7SS, being found at the cytoplasmic N-terminus of EccD [30], indicating that such proteins may be essential features of all T7SSs. To determine the subcellular location of EsaB-YFP, we blotted secreted and whole cell samples of the *esaB* mutant strain producing plasmid-encoded EsaB-YFP with the YFP antiserum. Fig. 3(a) shows that EsaB-YFP was associated exclusively with the cellular fraction.

We next fractionated these cells to obtain cytoplasm, cell wall and membrane fractions. Immunoblotting with antisera to control proteins known to localize to the cell membrane (SrtA) and cytoplasm (TrxA) indicated that the fractionation had been largely successful, although some SrtA was found in the cell wall fraction (Fig. 3b). Blotting these same fractions for the presence of EsaB-YFP showed that the protein localised to both the cytoplasm and membrane fractions. Some degradation of the fusion protein was also noted in these experiments which may result from the activation of proteases during fractionation. Indeed, in the membrane fraction it appears that EsaB-YFP migrated as a doublet band of around 37 kDa, indicating some probable truncation of the protein. When unfused YFP was produced in the wild-type strain it did not localise to the membrane (Fig. 3c), indicating that membrane binding was unlikely to be mediated through the YFP portion of the fusion.

Next we tested whether EsaB-YFP localised to the membrane through interactions with membrane components of the T7SS. To this end we repeated the fractionation in a strain carrying a chromosomal deletion in all twelve genes at the *ess* locus (Fig. 1a). However this did not alter the localisation of EsaB-YFP, which was still detected in both cytoplasm and membrane fractions (Fig. 3b). It is possible that EsaB-YFP localises to the membrane through interaction with additional membrane proteins. Alternatively, it may be that the membrane localisation arises as an artefact of the C-terminal YFP tag, since this tag is known to influence protein behaviour (e.g. [31]).

### Mutagenesis of conserved residues in EsaB

**Fig. 5.**
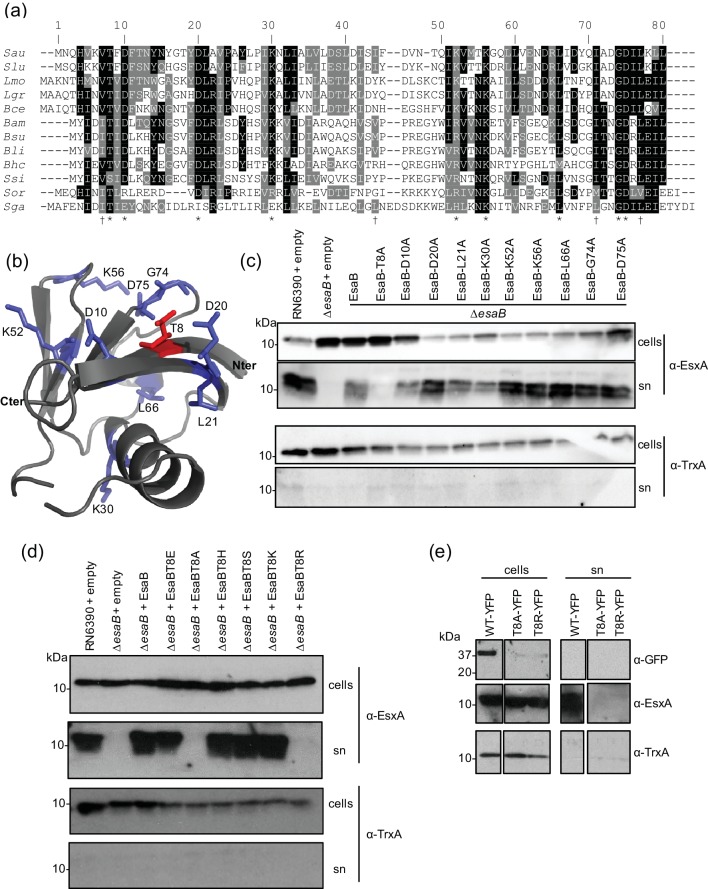
Site-directed mutagenesis of conserved residues of EsaB. (a) Sequence alignment of EsaB homologues from: Sau-*Staphylococcus aureus*; Slu-*Staphylococcus lugdunensis*; Lmo - *Listeria monocytogenes*; Lgr-*Listeria grayi*; Bce - *Bacillus cereus*; Bam-*Bacillus amyloliquefaciens*; Bsu-*Bacillus subtilis*; Bli-*Bacillus licheniformis*; Bhc-*Bhargavaea cecembensis*; Ssi-*Solibacillus silvestris*; Sor-*Streptococcus oralis*; Sga-*Streptococcus gallolyticus*. * indicate conserved residues and † indicates residues forming a potential hydrophobic patch that were mutated in this work. (b) Model of *S. aureus* EsaB with positions of conserved residues targeted for mutagenesis highlighted. The N- and C-termini are also indicated. (c) and (d) RN6390 harbouring empty pRAB11, and the isogenic *esaB* deletion strain harbouring pRAB11, or pRAB11 encoding native, the indicated variants of EsaB were cultured aerobically in TSB medium until an OD_600_ of 2 was reached. Samples were fractionated to give cells and supernatant (sn), and supernatant proteins were precipitated using TCA. For each gel, 10 µl of OD_600_1 adjusted cells and 15 µl of culture supernatant were loaded. Blots were probed with anti-EsxA, and anti-TrxA (cytoplasmic control) antisera. (e) The Δ*esaB* strain harbouring pRAB11 encoding EsaB-YFP (WT-YFP) or the T8A or T8R amino acid-substituted variants were cultured in TSB medium until mid-log phase and separated into cellular and supernatant fractions (sn). For each sample, 10 µl of OD_600_1 adjusted cells and 15 µl of culture supernatant were loaded and blots were probed with anti-EsxA, anti-TrxA or anti-GFP antisera. The cell samples and supernatant samples have been blotted on the same gels but intervening lanes have been spliced out.

An alignment of EsaB homologues encoded across firmicutes (Fig. 5a) identifies a number of highly conserved amino acids. Many of these are hydrophilic and fall on one face of the predicted structure of EsaB including T8 (*S. aureus* numbering) which is highly conserved as either threonine or serine, and the invariant K56. The presence of an invariant lysine is intriguing since there are a number of highly conserved lysine residues on the structurally-related protein ubiquitin, which are used to assemble polyubiquitin chains [32]. To probe potential roles of these conserved residues we mutated each of T8, D10, L21, K30, K52, K56, L66, G74 and D75 to alanine on plasmid-encoded EsaB and assessed whether the variant EsaB proteins were able to restore T7 secretion activity to the *esaB* deletion strain.

Fig. 5(c) shows that alanine substitutions of each of these conserved residues was tolerated by EsaB with the exception of T8A, which completely abolished EsaB activity. To test whether other side chain substitutions were permissive at T8, we subsequently constructed EsaB T8S, T8E, T8H, T8K and T8R substitutions. As seen in Fig. 5(d), in addition to T8A the T8R substitution also abolished EsaB activity, but the other substitutions resulted in active protein. Finally we attempted to assess whether the T8A and T8R inactivating substitutions altered the subcellular location of EsaB-YFP. However, when we introduced these into EsaB-YFP we found that they destabilised the protein as it was almost undetectable in whole cells (Fig. 5e), precluding further analysis. We are therefore unable to determine whether substitution of T8 directly alters EsaB function or has an indirect effect by disrupting folding.

## Discussion

In this work we have investigated the role of EsaB in Type VII secretion. EsaB proteins are conserved in firmicutes that produce the T7SS and are encoded at the same loci. Previous work had implicated EsaB in the regulation of *esxC* transcripts [11], although this cannot be a conserved role for EsaB proteins as they are found in all *S. aureus* strains, including the subset that do not encode *esxC* [16]. Here we show that EsaB does not regulate *esxC* in strain RN6390, nor any of the other genes encoded at the *ess* locus. Instead, deletion of *esaB* is associated with upregulation of genes involved in iron acquisition, mirroring the upregulation of iron-acquisition genes seen when the core T7 component, EssC, is absent [15]. This supports the notion that EsaB is an essential component of the secretion machinery in RN6390 that is necessary for activity, and in agreement with this, deletion of *esaB* prevented export of the T7-dependent extracellular proteins EsxA, EsxB and EsxC. This conclusion is also in agreement with related studies in *B. subtilis*, where the EsaB homologue YukD was shown to be essential for secretion of the WXG100 protein YukE [17, 18].

The precise role of EsaB in T7 secretion is unclear. Structural analysis of *B. subtilis* YukD shows that it shares a very similar fold to ubiquitin but that it lacks the ability to be conjugated with other proteins [20]. Interestingly, a structurally-related domain is associated with the actinobacterial T7SS, being found at the cytoplasmic N-terminus of the polytopic EccD membrane component [30], suggesting that EsaB-like domains may be essential features of all T7SSs. Given its small size and the observation that highly conserved residues fall primarily on one face of the protein, we reasoned that EsaB may interact with one or more components of the *S. aureus* T7SS, potentially regulating activity. Post-translational regulation of the *S. aureus* T7SS has been suggested because in some growth conditions the secretion machinery is present but there is no or very little substrate secretion [12, 19]. Other protein secretion systems are also post-translationally regulated, for example the flagellar Type III secretion system is regulated through interaction of the FliI component with the second messenger cyclic di-GMP [33], and Type VI secretion systems are regulated by phosphorylation [34]. In this context, EsaB proteins contain a highly conserved threonine (or serine) residue close to their N-termini which we considered as a potential site for phosphorylation. Intriguingly, substitution of EsaB T8 for alanine abolished the function of EsaB, although introduction of either the phospho-mimetic glutamate at this position or a positively charged lysine did not affect EsaB activity.

RNA-Seq analysis of the *esaB* mutant strain showed that in addition to a common set of genes showing similar regulation in the *esaB* and *essC* strains, a further subset of genes were uniquely deregulated in the *esaB* mutant. Many of the genes in this EsaB-specific subset are part of the AirSR regulon [26–29]. The AirSR two component system responds to oxidation signals via a redox-active [2Fe-2S] cluster in the sensor kinase AirS to regulate diverse sets of genes involved in anaerobic respiration, lactose metabolism and capsule biosynthesis. It is possible that these genes are dysregulated indirectly, for example in the absence of EsaB the T7 machinery may be in a state that causes ion leakage or membrane stress. Alternatively it is possible that EsaB interacts directly with a component of the Air system. In future it will be interesting to further decipher the roles of EsaB in T7 protein secretion and *S. aureus* physiology.

## Supplementary Data

101 Supplementary File 1
