# Supplementary material for: Functional analysis of the EsaB component of the Staphylococcus aureus Type VII secretion system
Source: Microbiology (Reading). 2017 Nov 22;163(12):1851–63. doi: 10.1099/mic.0.000580 (PMC5845737; doi:10.1099/mic.0.000580)
Supplement: Supplementary File 1 [file mic-163-1851-s001.pdf]

Functional analysis of the EsaB component of the *Staphylococcus aureus* Type VII secretion system

SUPPLEMENTARY INFORMATION

M. Guillermina Casabona[1], Grant Buchanan[1], Martin Zoltner[1], Catriona P. Harkins[2], Matthew T.G. Holden[2] and Tracy Palmer[1*]

[1]Division of Molecular Microbiology School of Life Sciences, University of Dundee, Dundee, UK.

[2]School of Medicine, University of St Andrews, St Andrews, KY16 9TF, UK.

*To whom correspondence should be addressed.

Tel +44 1382 386464

e-mail t.palmer@dundee.ac.uk

Primer	Nucleotide Sequence (5'-3')	Usage
EsaB-pET1	GCGCGC<u>CATATG</u>AATCAGCACGTAAAAGT AAC	Amplification of *esaB* synthetic gene for cloning into pET15b
EsaB-pET2	GCGCGCCTCGAGTCACAGCAGTTTCAGAA TATCGCCATC	Amplification of *esaB* synthetic gene for cloning into pET15b
EsaB-fw	TTTT<u>GTTAAC</u>TCATAAAGGGAGACGAACG	Amplification of *esaB* gene and its own RBS from RN6390 genome for cloning in pRAB11
EsaB-rev	TTA<u>GAATTCC</u>TCCTATAGTAACTTCAAAATA TC	Amplification of *esaB* gene and its own RBS from RN6390 genome for cloning in pRAB11
MGC3	CACGTAAAAGTAGCATTTGATTTTACT	Quick change mutagenesis EsaB T8A
MGC4	AGTAAAATCAAATGCTACTTTTACGTG	Quick change mutagenesis EsaB T8A
MGC153	CACGTAAAAGTAAAATTTGATTTTACT	Quick change mutagenesis EsaB T8K
MGC154	AGTAAAATCAAATTTTACTTTTACGTG	Quick change mutagenesis EsaB T8K
MGC161	CACGTAAAAGTAAGATTTGATTTTACT	Quick change mutagenesis EsaB T8R
MGC162	AGTAAAATCAAATCTTACTTTTACGTG	Quick change mutagenesis EsaB T8R
MGC163	CACGTAAAAGTACATTTTGATTTTACT	Quick change mutagenesis EsaB T8H
MGC164	AGTAAAATCAAAATGTACTTTTACGTG	Quick change mutagenesis EsaB T8H
MGC53	CACGTAAAAGTAGAATTTGATTTTACT	Quick change mutagenesis EsaB T8E
MGC54	AGTAAAATCAAATTCTACTTTTACGTG	Quick change mutagenesis EsaB T8E
MGC174	CAGCACGTAAAAGTATCATTTGATTTTACT	Quick change mutagenesis EsaB T8S
MGC175	AGTAAAATCAAATGATACTTTTACGTGCTG	Quick change mutagenesis EsaB T8S
MGC5	AAAGTAACATTTGCATTTACTAATTAT	Quick change mutagenesis EsaB D10A
MGC6	ATAATTAGTAAATGCAAATGTTACTTT	Quick change mutagenesis EsaB D10A
MGC7	TACGGCACATATGCATTAGCAGTACCA	Quick change mutagenesis EsaB D20A
MGC8	TGGTACTGCTAATGCATATGTGCCGTA	Quick change mutagenesis EsaB D20A
MGC133	GGCACATATGACGCAGCAGTACCAGCA	Quick change mutagenesis EsaB L21A
MGC134	TGCTGGTACTGCTGCGTCATATGTGCC	Quick change mutagenesis EsaB L21A
MGC9	TATTTACCGATAGCAAACTTAATAGCT	Quick change mutagenesis EsaB K30A
MGC10	AGCTATTAAGTTTGCTATCGGTAAATA	Quick change mutagenesis EsaB K30A
MGC11	AATACACAAATTGCAGTGATGACGAAA	Quick change mutagenesis EsaB K52A
MGC12	TTTCGTCATCACTGCAATTTGTGTATT	Quick change mutagenesis EsaB K52A
MGC13	AAAGTGATGACGGCAGGTCAATTACTT	Quick change mutagenesis EsaB K56A

MGC14	AAGTAATTGACCTGCCGTCATCACTTT	Quick change mutagenesis EsaB K56A
MGC15	GAAAATGATCGAGCAATTGATTATCAA	Quick change mutagenesis EsaB L66A
MGC16	TTGATAATCAATTGCTCGATCATTTTC	Quick change mutagenesis EsaB L66A
MGC135	CAAATCGCTGATGCAGATATTTTGAAG	Quick change mutagenesis EsaB G74A
MGC136	CTTCAAAATATCTGCATCAGCGATTTG	Quick change mutagenesis EsaB G74A
MGC17	ATCGCTGATGGAGCAATTTTGAAGTTA	Quick change mutagenesis EsaB D75A
MGC18	TAACTTCAAAATTGCTCCATCAGCGAT	Quick change mutagenesis EsaB D75A
region-1-f	CAGGAGGTTTCTAGTTATGGC	RT-PCR, region 1
region-1-r	GTTCTTGAACGGCATCAGC	RT-PCR, region 1
region-2-f	GCATATGTACGCAAAGTAGGAC	RT-PCR, region 2
region-2-r	TCGTTAGTTGCTCTTGAGTTC	RT-PCR, region 2

Table S1. Oligonucleotides used in this study. Restriction enzyme sites have been underlined.
